# Study on hydration mechanism and environmental safety of thermal activated red mud-based cementitious materials

**DOI:** 10.1007/s11356-023-25832-w

**Published:** 2023-03-11

**Authors:** Junge Zhu, Hongzhi Yue, Laijun Ma, Zichao Li, Rong Bai

**Affiliations:** College of Materials Science and Engineering, Shandong University of Technology, Zibo, 255000 Shandong China

**Keywords:** Thermal activated red mud, Steel slag, Hydration mechanism, Solidified of alkali and heavy metals

## Abstract

Red mud (RM) cementitious materials were prepared with the thermally, thermoalkali- or thermocalcium-activated RM, steel slag (SS), and other additives. The effects of different thermal RM activation methods on the cementitious material hydration mechanisms, mechanical properties, and environmental risks were discussed and analyzed. The results showed that the hydration products of different thermally activated RM samples were similar with the main products being C-S–H, tobermorite, and Ca(OH)_2_. Ca(OH)_2_ was mainly present in thermally activated RM samples, and the tobermorite was mainly produced by samples prepared with thermoalkali- and the thermocalcium-activated RM. The mechanical properties of the samples prepared by thermally and thermocalcium-activated RM had early-strength properties, while the thermoalkali-activated RM samples were similar to the late-strength type of cement properties. The average flexural strength of thermally and the thermocalcium-activated RM samples at 14 days were 3.75 MPa and 3.87 MPa respectively, whereas, the 1000 °C thermoalkali-activated RM samples only at 28 days was 3.26 MPa; the above data could reach the single flexural strength (3.0 MPa) of the first-grade pavement blocks of the building materials industry standard of the People’s Republic of China-concrete pavement blocks (JC/T446-2000). The optimal preactivated temperature for different thermally activated RM was different; the optimal preactivated temperature for both thermally and thermocalcium-activated RM was 900 °C, and the flexural strength was 4.46 MPa and 4.35 MPa, respectively. However, the optimal preactivated temperature of thermoalkali activated RM at 1000 °C. The 900 °C thermally activated RM samples had better solidified effects for heavy metal elements and alkali substances. 600~800℃ thermoalkali activated RM samples had better solidified effects for heavy metal elements. Different temperatures of thermocalcium-activated RM samples showed different solidified effects on different heavy metal elements, which may be due to the influence of thermocalcium activation temperature on the structural changes of the hydration products of the cementitious samples. In this study, three thermal RM activation methods were proposed, and the co-hydration mechanism and environmental risk study of different thermally activated RM and SS were further elucidated. This not only provides an effective method for the pretreatment and safe utilization of RM, but also facilitates the synergistic resource treatment of solid waste and further promotes the research process of replacing part of traditional cement with solid waste.

## Introduction

Industrial solid waste has always been the top priority of environmental management. “Waste for waste” is to create the technology to transform waste into value-added products (Azevedo Afonso et al. 2020). With the promotion of industrial modernization, the number of alumina manufacturing plants is increasing, resulting in the accumulation of the waste residue—red mud (RM), rising annually. RM contains alkaline substances and some heavy metals; its perennial accumulation will not only occupy a large amount of land, but also lead to land alkalization and water pollution (Nikbin et al. 2017). Additionally, the treatment cost of RM is high, which makes its recovery rate low. The application of RM to building materials will greatly improve its utilization rate. To reduce energy consumption and improve environmental performance, RM-based cementitious materials have become a research hotspot.

RM contains Ca, Al, and Si substrates, which have the potential to prepare cementitious materials. Because of its low activity, the single RM is difficult to prepare a good performance of cementitious material. SS contains aluminosilicate minerals with a higher SiO_2_/Al_2_O_3_ ratio, which can provide complementary gelling activity factors for RM (Qaidi Shaker et al. 2022). Many studies have focused on the synergistic hydration reaction between RM and SS. In order to generate hydration products such as C-S–H and AFt, RM, SS, and electrolytic manganese slag were co-hydrated to produce the cementitious material with unconfined compressive strength of 5.48 MPa at 7 days, bending tensile strength of 1.80 MPa and indirect tensile strength of 0.60 MPa at 90 days, which met the strength requirements of primary highway pavement base (Tan et al. 2021). The addition of SS could improve the mechanical strength of the RM-blast furnace slag (BFS) system, and the synergistic reaction could promote the formation of ettringite and calcium aluminate hydrate, showing that the 28-day compressive strength of the sample could reach 12.78 MPa, with an increase of 59.84% (Li et al. 2021). RM, SS, and desulfurized gypsum (DG) could produce additional hydration products to make the cementitious materials compact, because the alkali in RM and the sulfate in DG could stimulate the activity of SS, thus promoting the cementitious hydration reaction (Hao et al. 2022). The hydration products of flocculation and flake crystals appeared in the cementitious products prepared by RM, SS, and cement clinker. The mechanical properties of the sample were poor, because the addition of RM exceeded the scope of stabilizing the hydration products, and its low activity hindered the hydration process in the system, which implied that the addition amount of RM had a crucial effect on the overall performance of the cementitious material (Liang et al. 2019). A large amount of RM is of great significance to its resource utilization. The content of RM would significantly affect the morphology of hydration products of cementitious materials and further affect the mechanical properties. Therefore, the preactivated of RM should be considered on the premise of not reducing the amount of RM added. The activation of RM promoted the hydration process of the cementitious material samples and also increased the activities of Ca^2+^, Si^2+^, and Al^3+^. RM was directly calcined to destroy the structure of silicon and aluminum matrix in the RM phase (Kenne Diffo et al. 2015), and the calcined RM formed a metastable network structure of aluminosilicates, showing similar properties to volcanic ash (Bayat et al. 2018). The thermoalkali activation of RM could increase the amorphous structure of its particles and thus improve its gelling activity.

The cementitious materials prepared in the above study were all completed with less than 50% RM with SS and other solid waste materials. There were few studies on the co-preparation of cementitious materials only using RM and SS, and the environmental risk of cementitious materials had not been properly studied. Combined with the above problems, this paper proposed to use RM, thermally activated RM, thermoalkali-activated RM, and thermocalcium-activated RM and SS to prepare cementitious materials on the premise of ensuring that the RM content was higher than 50% and to explore the influence of different thermally activated RM on the mechanical properties, hydration mechanism, and environmental safety of cementitious materials. Considering the characteristics of high-energy consumption and the unfriendly environment of traditional cement, this paper introduced the use of RM and SS to prepare cementitious materials that can replace traditional cement, which provided a new research perspective and idea for the resource utilization of RM and SS, and also promoted the research process of replacing traditional cement with solid waste cementitious materials.

## Materials and methods

### Raw materials

**Fig. 1:**
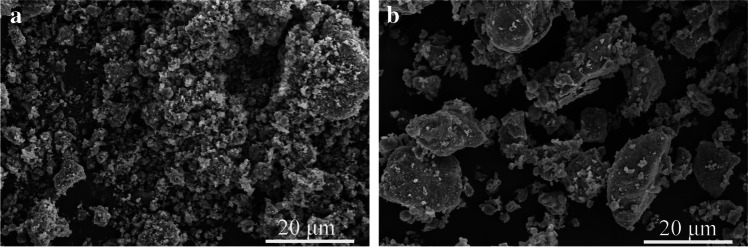
Microstructure analysis of raw materials (**a** RM, **b** SS)

**Fig. 2:**
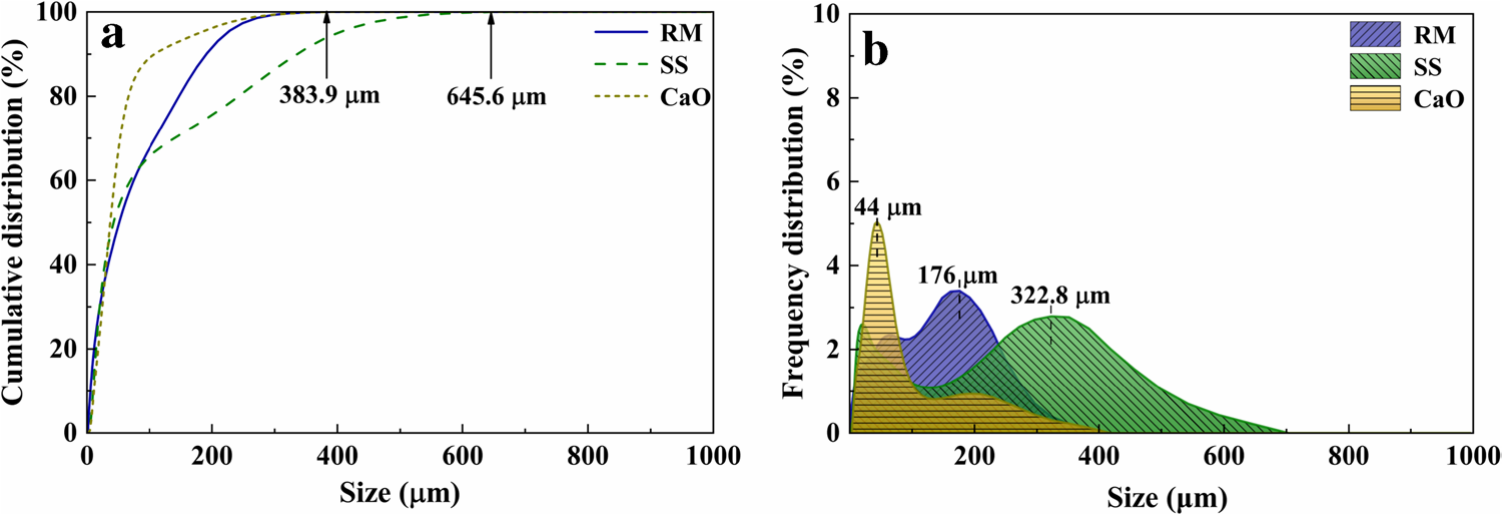
Particle size analysis of raw materials (**a** the cumulative distribution of raw materials, **b** the frequency distribution of raw materials)

**Fig. 3:**
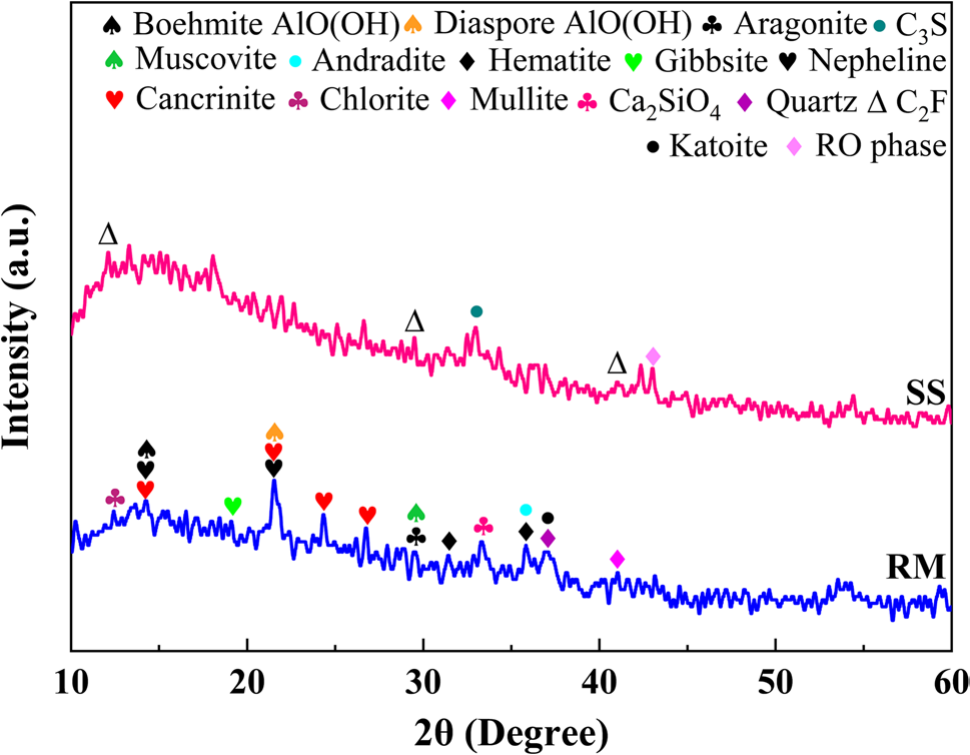
XRD spectra of raw materials

The RM was provided by the Aluminum Corporation of China Shandong Branch; the SS used in this research was from Laiwu Iron and Steel Group Co., Ltd. Analytically, pure CaO and sodium hydroxide were purchased from Tianjin Zhiyuan Chemical Reagents Co., Ltd. The concentration of sodium silicate solution was 0.5625 (the mass ratio of sodium silicate analysis of pure: deionized water = 1.6875:3). The microscopic morphology, particle size analysis, chemical composition, and mineral phase composition of raw materials are respectively shown in Fig. 1, Fig. 2, Table 1, and Fig. 3.

**Table 1:** Chemical composition of raw materials (wt%)

Materials	Al_2_O_3_	SiO_2_	Fe_2_O_3_	TiO_2_	Na_2_O	MgO	CaO	MnO	P_2_O_5_	SO_3_	K_2_O
RM	12.2	11.1	30.9	2.65	6.27	0.832	1.75	0.149	0.113	0.593	0.144
SS	6.01	18.9	19.4	1.01	0.413	5.39	34.5	3.10	1.95	0.298	0.385

Figure 1 displays the microstructure of RM and SS. Combined with Fig. 2, it can be seen that the particle size of RM is finer, less than 383.09 µm, and the proportion of particles at 176 µm is the largest. The RM is mutually bonded and aggregated, and the main morphology is spherical and flake. SS is an obvious ribbed irregular particle with a particle size lower than 645.6 µm and the largest proportion of particles with a size of 322.8 µm. The particle size of CaO occupies the largest proportion at 44 µm. Table 1 shows that the contents of Al_2_O_3_, SiO_2_, and CaO in the RM were 12.2%, 11.1%, and 1.75%, respectively. The contents of Al_2_O_3_, SiO_2_, and CaO in the SS were 6.01%, 18.9%, and 34.5%, respectively, which indicate that the effective cementitious components in RM and SS could act in concert. Mineralogically, the main phases were nepheline (NaAlSiO_4_), quartz (SiO_2_), cancrinite((Na, Ca, K)_7–8_[(Si, Al)_12_O_24_](CO_3_, OH)_2_‧2 ~ 3H_2_O), hematite (Fe_2_O_3_), mullite (3Al_2_O_3_‧2SiO_2_), aragonite (CaCO_3_), boehmite AlO(OH) (Hajjaji et al. 2013), and katoite (Ca_2.93_Al_1.97_Si_0.64_O_2.56_(OH)_9.44_) in the RM and C_2_F, C_3_S, and RO phases (MgO, FeO, MnO, and f-CaO) in the SS. In the alkaline environment provided by RM, silicon and aluminum compounds react with calcium hydroxides to form cementitious materials.

### The experimental process

RM and SS, which were the main raw materials, were sieved (100 mesh), dried, ball-milled, and stored. Three equal parts of RM were taken out and put into an energy-saving box furnace (SX-G80133), respectively, for thermal activation, thermoalkali activation, and thermocalcium activation. Three thermal activations were set the same temperature gradient (600 °C, 800 °C, 900 °C, 1000 °C), respectively, denoting R6 ~ R10, RA6 ~ RA10, and RC6 ~ RC10. The NaOH and CaO used for thermoalkali activation and thermocalcium activation were analytically pure reagents used in the laboratory (the mass ratio of NaOH or CaO: RM = 1:9). The setting of RM preactivated temperature and duration was shown in Table 2. CaO and the sodium silicate solution were added. The detailed sample formula was shown in Table 3. The sample was prepared according to a water-cement ratio of 0.3 ~ 0.5 (The amount of deionized water added to each group of samples was shown in Table 4. Deionized water was selected to eliminate the interference of other ions in the analysis of heavy metal ion migration regularity).

**Table 2:** The heating schedule of RM

Initial temperature/°C	Temperature of termination/°C	Heating rate °C/min	Heating time/min	Holding time/min
Room temperature	600 °C	3.3	182	30
Room temperature	800 °C	3.3	243	30
Room temperature	900 °C	3.3	273	30
Room temperature	1000 °C	3.3	303	30

The heating time error was set to ± 1 min

**Table 3:** Sample formula table

Mix name	Proportion (wt%)			
A	RM	SS	CaO	Admixture
	60	30	10	4.5
B_1_	R6	SS	CaO	Admixture
	60	30	10	4.5
B_2_	R8	SS	CaO	Admixture
	60	30	10	4.5
B_3_	R9	SS	CaO	Admixture
	60	30	10	4.5
B_4_	R10	SS	CaO	Admixture
	60	30	10	4.5
C_1_	RA6	SS	CaO	Admixture
	60	30	10	4.5
C_2_	RA8	SS	CaO	Admixture
	60	30	10	4.5
C_3_	RA9	SS	CaO	Admixture
	60	30	10	4.5
C_4_	RA10	SS	CaO	Admixture
	60	30	10	4.5
D_1_	RC6	SS	CaO	Admixture
	60	30	10	4.5
D_2_	RC8	SS	CaO	Admixture
	60	30	10	4.5
D_3_	RC9	SS	CaO	Admixture
	60	30	10	4.5
D_4_	RC10	SS	CaO	Admixture
	60	30	10	4.5

R6, R8, R9, and R10: RM thermal activation at 600 °C, 800 °C, 900 °C, and 1000 °C; RA6, RA8, RA9, and RA10: RM thermoalkali activation at 600 °C, 800 °C, 900 °C, and 1000 °C; RC6, RC8, RC9, and RC10: RM thermocalcium activation at 600 °C, 800 °C, 900 °C, and 1000 °C

**Table 4:** Water-cement ratio distribution table of sample

Samples	Powder/g	Mixing water/g	Water-cement ratio
A	1500	495	0.33
B	1500	585	0.39
C	1500	750	0.50
D	1500	630	0.42

Mixing water: deionized water

The slurry was mixed separately according to the above ratios (three copies per group) and placed in a standard sand mold (40 mm × 40 mm × 160 mm). According to the standard curing conditions for concrete (temperature 20 °C ± 1 °C, relative humidity ≥ 90%), the material was cured for 24 h, demolded, and then further cured for 3 days, 7 days, 14 days, and 28 days, respectively. Finally, the samples that were cured for 28 days were dried, broken, and ground for testing. Based on the acetic acid buffer solution method (HJ/T 300–2007), an extraction agent (2^#^) was selected, and the thermally activated RM powder and cementitious sample blocks were soaked for 1 day, 7 days, 15 days, and 30 days, respectively, and the leaching solution was collected at these timepoints and stored for testing.

### Testing conditions

**Fig. 4:**
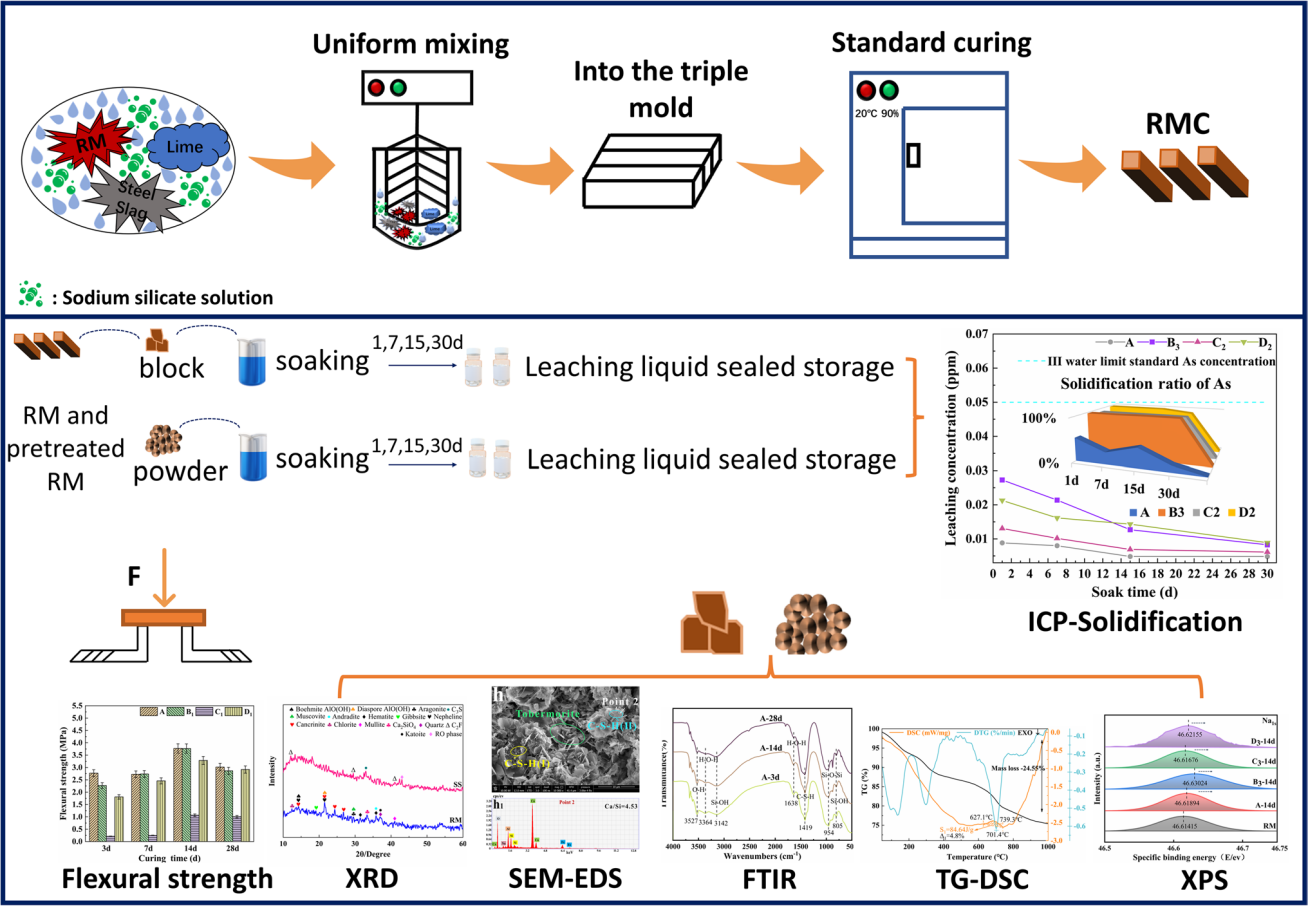
Flow chart of preparation and detection of RMC

After the samples reached the end of their fixed curing periods (3 days, 7 days, 14 days, and 28 days), they were removed from the standard curing box and analyzed for flexural strength (WDW-20 electronic universal testing machine) and investigated via XRD (AXS-D8-02), scanning (SEM–EDS Quanta 250), FT-IR (Nicolet 5700), and XPS analysis (Thermo Scientific ESCALAB Xi +). The leaching liquid from the 28-day sample was analyzed with ICP-MS 7500ce detection (Agilent 7500ce-Environmental type) (Fig. 4).

## Results and discussion

### Mechanical properties

**Fig. 5:**
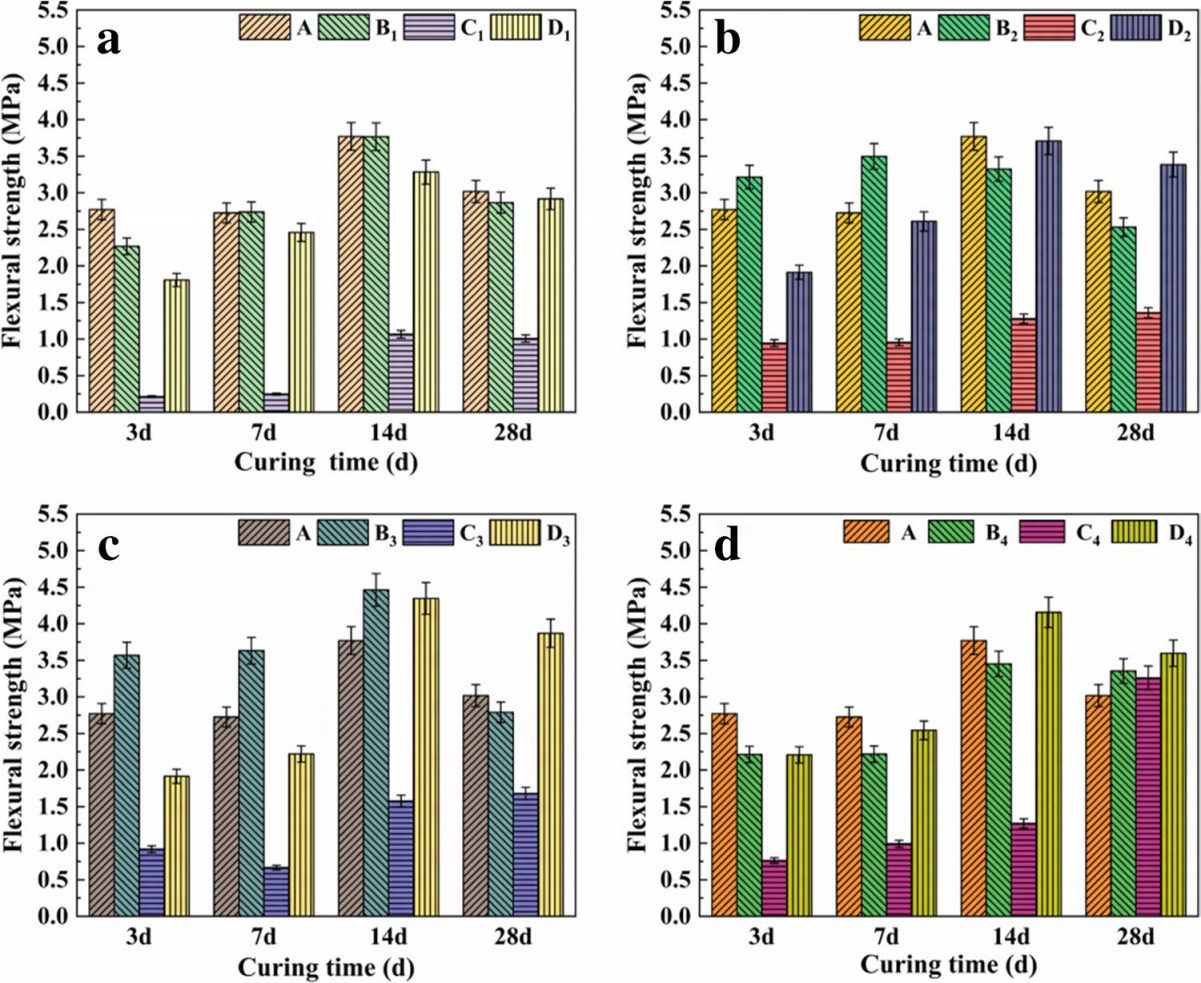
The flexural strength of samples at 3, 7, 14, and 28 days (**a** the samples of RM and preactivated RM at 600 °C, **b** the samples of RM and preactivated RM at 800 °C, **c** the samples of RM and preactivated RM at 900 °C, **d** the samples of RM and preactivated RM at 1000 °C)

The changes in the flexural strength at 3, 7, 14, and 28 days with different pretreated RMs are shown in Fig. 5. The flexural strength of the samples showed an increasing trend from 3 to 14 days, and the maximum flexural strengths of A, B_3_, and D_3_ at 14 days were 3.8 MPa, 4.5 MPa, and 4.3 MPa, respectively. However, the maximum flexural strength of group C samples was 3.3 MPa which occurred in the C_4_-28-day sample, indicating that alkali treatment of RM at 1000 °C could promote its hydration process, while pretreatment and calcium treatment of RM at 900 °C could improve the early hydration rate of the sample. The alkali in RM could enhance the hydration activity of the matrix by dissolving Si and Al in the SS and reacting to form a hydrated sodium silicate gel (Xu et al. 2021; Liu et al. 2021). After pretreatment, the RM activity factor was activated, and the RM further promoted the hydration reaction with SS in an alkaline environment to produce hydration products such as C-S–H. However, if only the content of alkali substances was increased without proper adjustment of the Ca matrix, it would cause the lower early strength of the samples (Nunes and Borges 2021; Liu et al. 2019), which was the reason for the lower early strength of the samples after thermoalkali activation. According to the “Concrete Pavement Brick Building Materials Industry Standard of the People’s Republic of China” (JC/T446-2000), the flexural strength of groups A, B, and D at 14 days and C_4_ at 28 days could meet the single flexural strength of first-grade pavement bricks (≥3.0 MPa).

### XRD phase analysis

**Fig. 6:**
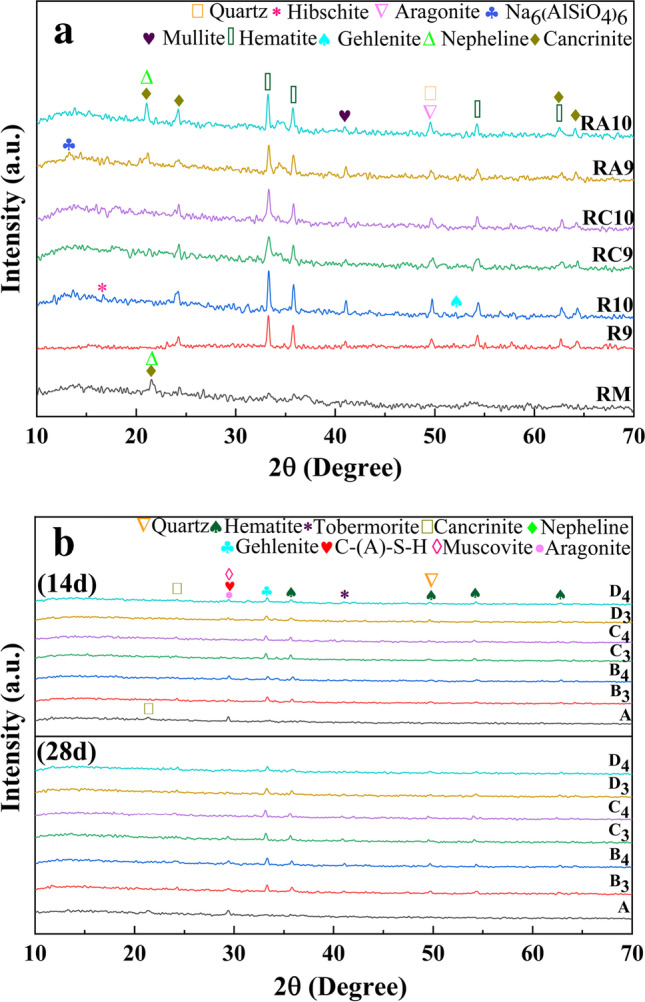
XRD phase analysis of activated RM and samples (**a** XRD analysis of raw materials, **b** XRD analysis of samples at 14 days and 28 days)

Figure 6 a shows the phases in RM before and after activation. It demonstrates that cancrinite ((Na, Ca, K)_7–8_[(Si, Al)_12_O_24_] (CO_3_, OH)_2_‧2 ~ 3H_2_O)) (at approximately 24°) and hematite (Fe_2_O_3_) (at 34°, 36°, and 54°) levels increased in the activated material. Meanwhile, mullite (3Al_2_O_3_‧2SiO_2_) (at 41°) and aragonite (CaCO_3_) (at 49°) appeared in it. It is worth noting that there was no noticeable phase peak at 24° in R9, R10, RC9, and RC10. This result indicated that the phase composition of silicate, aluminate, and inorganic calcium in the activated material was greater than that before activation.

Fig. 6b demonstrated that the cementitious samples with cured for 14d and 28d have similar phases. In addition to the hematite (Fe_2_O_3_) and quartz (SiO_2_) already present in the raw material, there were hydration products that included C-A-S–H and muscovite (3Al_2_O_3_‧2SiO_2_), and gehlenite (C_2_AS) appeared in the samples. The mullite (3Al_2_O_3_‧2SiO_2_) (42°) in the raw material changed to tobermorite (Ca_5_Si_6_O_16_(OH)_2_‧4H_2_O) in the samples.

By combining Fig. 5 and Fig. 6, it can be seen that the changes in the phases of samples prepared by different pretreatment methods were similar, but there were also differences. The samples of groups A, B, C, and D had tobermorite (Ca_5_Si_6_O_16_(OH)_2_‧4H_2_O), aragonite (CaCO_3_), and C-(A)-S–H. Samples B, C, and D had gehlenite (C_2_AS) and cancrinite ((Na, Ca, K)_7~8_ [(Si, Al)_12_O_24_] (CO_3_, OH)_2_‧2 ~ 3H_2_O)). These phases did not exist in group A. The above phenomenon demonstrated that relative un-activated RM, sodium aluminosilicate was produced by the thermal activation of RM, α-dicalcium silicate and superbasic sodium aluminosilicate were generated by the thermoalkali activation of RM, and calcium aluminosilicate formed via that thermocalcium activation of RM, and these could be collectively referred to as active aluminosilicate.

### Scanning analysis (SEM and EDS)

The samples obtained by different activation methods had unique microscopic characteristics for different curing periods. Since the treatment of RM with alkali at 1000 °C resulted in better mechanical properties at 28 days and promoted the hydration reaction of cementitious samples, A, B_4_, C_4_, and D_4_ were selected for discussion to further describe the microstructures of the hydration products of RM samples with different pretreatments at 1000 °C. Combined with the XRD spectrum, it can be seen that tobermorite, an important hydration product, was produced in the cementitious samples before and after activation. The above finding indicated that the formulation of an RM-based cementitious system is feasible. However, the morphologies of the hydration products obtained by different activation methods were different.

**Fig. 7:**
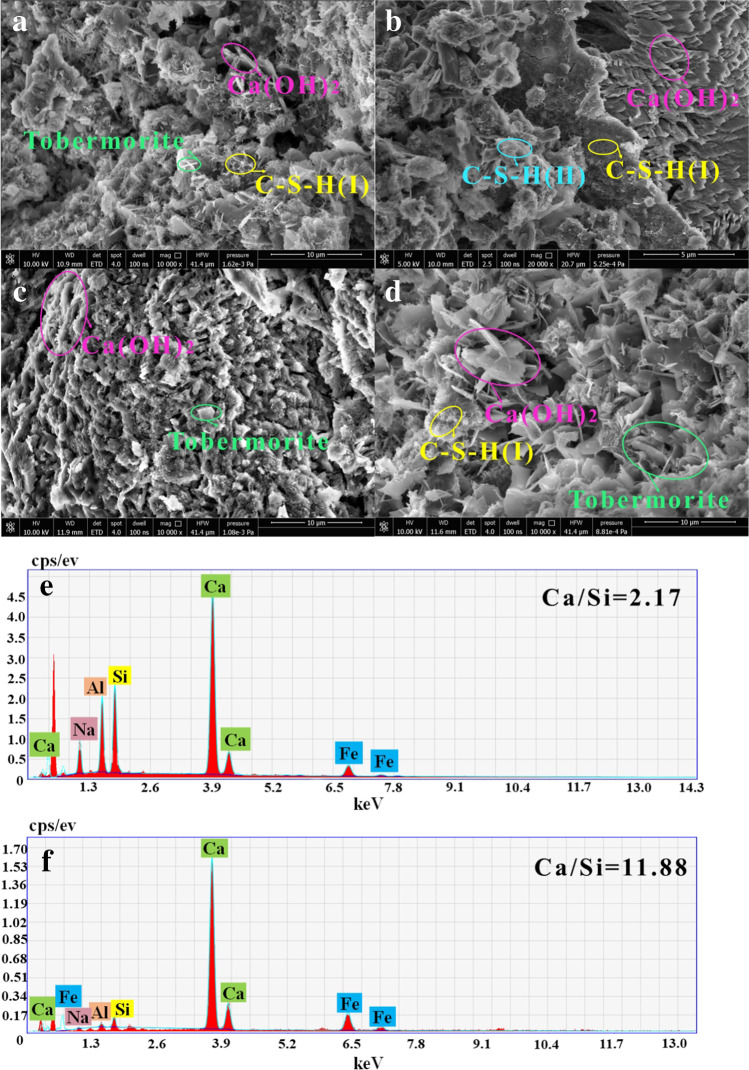
The SEM–EDS surface micrograph of samples at 14 days (**a** RM-based cementitious sample, **b** 1000 °C RM thermal activation, **c:** 1000 °C RM thermoalkali activation, **d** 1000 °C RM thermocalcium activation, **e** EDS surface of C_4_, **f** EDS surface of D_4_)

Figure 7 indicates that the hydration products C-S–H and tobermorite were produced in the microstructure of the samples at 14 days. Tobermorite containing a uniform appearance appeared in D_4_. Figure 5 and Fig. 7 collectively show that the flexural strength of D_4_ was relatively high, which indicated that the hydration reaction of D_4_ had progressed to a sufficient degree at the age of 14 days. The hydration product distribution uniformity was in the order D_4_ > B_4_ > C_4_ > A. Based on the analysis of Fig. 7 c, d–f, it can be seen that the hydration products in C_4_ were mostly loose structures with small needle-like and flake-like crystals of tobermorite. In D_4_, the tobermorite content was high and evenly distributed. Combined with EDS analysis, it can be seen that the content of Ca and Si in C_4_ was higher than that in D_4_, which indicated that D_4_ (at 14 days) had reached a better hydration state. Therefore, its strength at this early timepoint was higher than that of the other groups. The content of Si in C_4_ was higher, which was beneficial for promoting the subsequent hydration reaction.

**Fig. 8:**
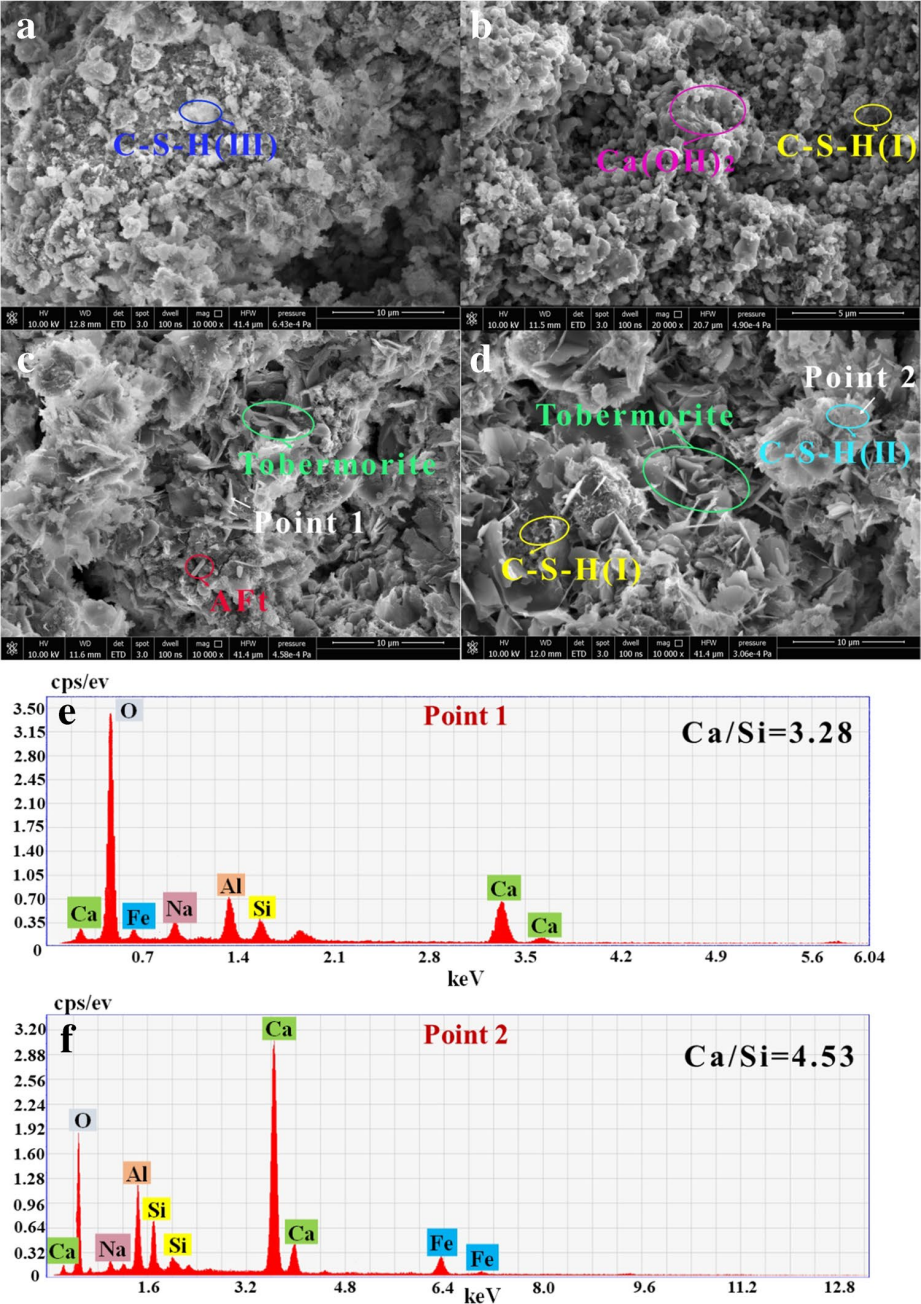
The SEM–EDS point micrograph of samples at 28 days (**a:** RM-based cementitious sample; **b:** 1000 °C RM thermal activation; **c:** 1000 °C RM thermoalkali activation; **d:** 1000 °C RM thermocalcium activation; **e:** EDS point of C_4_; **f:** EDS point of D_4_)

Figure 8 demonstrates that the microstructure of sample A at 28 days was irregular and loose, with few hydration products, indicating that the hydration reaction of the RM sample had stopped at 28 days, which was also related to the low activity of SS. This proved that the Ca and Si contained in the un-activated RM could not provide sufficient hydration conditions. A large number of small round lamellae (Ca(OH)_2_) (Chen et al. 2019; Pan et al. 2002; Kong et al. 2018) and locally opaque clumps (C-S–H-I) appeared in B_4_ at 28 days. C_4_ showed distinct spicules and even a small number of rods, and D_4_ showed more acicular and lamellar interwoven structures.

By combining with Fig. 7 and Fig. 8, it could be determined that the samples that were not activated, thermally activated at 1000 °C, and thermocalcium activated at 1000 °C showed an early rapid setting phenomenon, which revealed that the strength of the above samples originated from a direct hydration reaction. The strength that developed at early timepoints through the hydration reaction of un-activated RM originated from the fact that the Ca hydroxide in RM could be easily converted into calcium carbonate. Nevertheless, the sample activated by thermoalkali at 1000 °C had a higher Si content, and the release ratio of alkali in the RM after thermoalkali activation was relatively low. In the sample, Si reacted with the OH^−^ that was released from the RM to form a silicon-oxygen tetrahedra, so the hydration reaction was reflected in the later stages. It is worth noting that in these four groups of samples, the microstructure of C-S–H changed, which was related to the content of Ca and Si. The gelation hydration equation of the samples is as follows:

1 $$\mathrm{CaO}+{\mathrm{H}}_{2}\mathrm{O}\to \mathrm{Ca}{\left(\mathrm{OH}\right)}_{2}$$

2 $$\mathrm{Ca}{\left(\mathrm{OH}\right)}_{2}+{\mathrm{SiO}}_{2}\to {\mathrm{Ca}}_{5}{\mathrm{Si}}_{6}{\mathrm{O}}_{16}(\mathrm{OH})\cdot{4\mathrm{H}}_{2}\mathrm{O}(\mathrm{Tobermorite})$$

3 $$3{\mathrm{CaO \cdot Al}}_{2}{\mathrm{O}}_{3}\left({\mathrm{C}}_{3}\mathrm{A}\right)+{\mathrm{CaSO}}_{4}+\mathrm{CaO}\to \mathrm{C}-\mathrm{S}-\mathrm{H}(\mathrm{I}-\mathrm{II}-\mathrm{III})$$

4 $$3{\mathrm{CaO\cdot Al}}_{2}{\mathrm{O}}_{3}\cdot{6\mathrm{H}}_{2}\mathrm{O}\left({\mathrm{C}}_{3}{\mathrm{AH}}_{6}\right)+3\left({\mathrm{CaSO}}_{4}\cdot {2\mathrm{H}}_{2}\mathrm{O}\right)+{6\mathrm{H}}_{2}\mathrm{O}\to \mathrm{Aft}$$

The reaction equation showed that the hydration products could be collectively referred to as C-S–H. Tobermorite crystals were the main crystal phases of the sample in this work, which was closely related to the mechanical properties of the sample. However, C-S–H in Eq. (3) could be divided into I-II-III due to the differences in morphologies, and the differences in morphologies were related to the content of Ca and Si. The mass ratio of Ca/Si was further calculated by combining with Fig. 7 f and Fig. 8 f. When the mass ratio of Ca/Si was 11.88, the morphology of the hydration product was in the form of a fuzzy fiber honeycomb crystal (C-S–H-I). When the mass ratio of Ca/Si was 4.53, the morphology of the hydration products was in the form of a translucent crystal with acicular inclusion (C-S–H-II). When the mass ratio of Ca/Si was less than 4, the structure of the hydration products was in the form of an opaque floc (C-S–H-III). The results demonstrated that a low mass ratio of Ca/Si was unfavorable to the formation of tobermorite crystals. The high mass ratio of Ca/Si promoted the conversion of aluminum oxide tetrahedra to aluminum oxide hexahedra, which inhibited the reaction of the aluminum oxide tetrahedra and silicate and the formation of C-A-S–H. Generally, the activation effects of these three kinds of thermal activation methods were distinct. The effect of thermoalkali activation was opposite to that of the other thermal activation methods, which further indicated that thermoalkali activation could effectively stimulate the Si^2+^ in RM. This was beneficial for the later hydration reaction. Thermal activation and thermocalcium activation provided the conditions for the early hydration reaction.

### FT-IR analysis

**Fig. 9:**
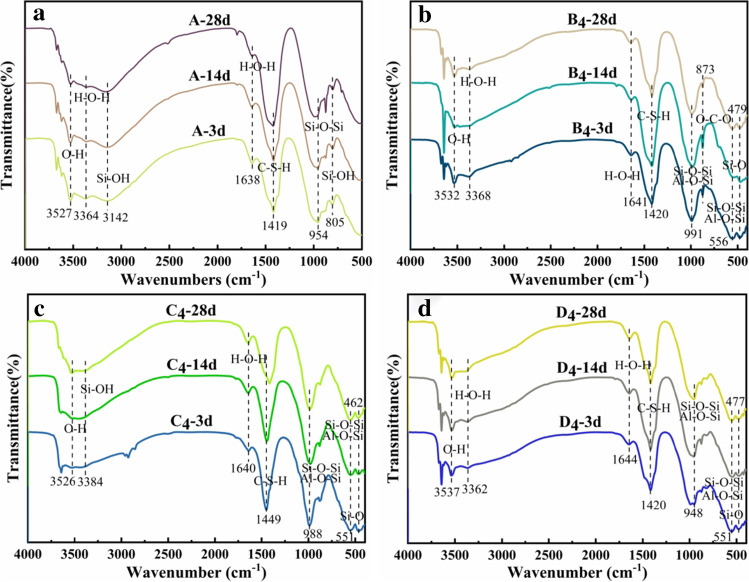
FT-IR spectra of samples at 3, 14, and 28 days (**a** RM-based cementitious sample, **b** 1000 °C RM thermal activation, **c** 1000 °C RM thermoalkali activation, **d** 1000 °C RM thermocalcium activation)

Figure 9 shows the infrared spectra of the four groups of samples prepared by different activation methods over different curing periods. The spectra of the four groups were similar, with similar absorption bands except for differences in absorption intensities.

Figure 9 a shows that the peak at 805 cm^−1^ represented the bending vibration of Si–OH, which existed throughout the 28-day curing cycle. This signified that the Si–OH in the hydration product C-S–H was in an unstable state and did not form a stable structure with the other groups. The absorption peak at 1419 cm^−1^ indicated the emergence of C-S–H, and the absorption peak at 954 cm^−1^ was the asymmetric stretching vibration band of Si–O-Si in C-S–H. The peaks at 1638 cm^−1^ and 3364 cm^−1^ exhibited the bending vibration of water (H–O-H). The broad absorption peak at 3142 cm^−1^ was the stretching vibration of Si–OH, which together with the bending vibration of Si–OH at 805 cm^−1^, represented the hydration state of the Si–OH group. The peak at 3142 cm^−1^ was a group of Si–OH bonds that had not been adequately polymerized during the gelation hydration reaction. The Si–OH group at 805 cm^−1^ represented the free-state Si–OH groups in the samples. There was a narrow absorption peak for O–H at 3527 cm^−1^.

By comparing Fig. 9 b, c, and d, in which the RM was treated by different activation methods, it can be seen that the wavenumber for C-S–H was consistently in the range of 1419 ~ 1449 cm^−1^, but the bending vibration of Si–O occurred at 462 ~ 479 cm^−1^, which was the characteristic spectral band for quartz. The Si–OH group in Fig. 9 a was at 805 cm^−1^, whereas it disappeared in Fig. 9 b, c, and d. The broad absorption peak for Si–O at 3142 cm^−1^ in Fig. 9 a was only found in Fig. 9 c, and the wavenumber position shifted to 3384 cm^−1^, which indicated that after thermal activation and thermocalcium activation, the number of free Si–OH groups decreased, and most of the Si–OH groups were consumed by the gelation hydration reaction, which increased the degree of polymerization in the hydration products. Figure 9 b demonstrates the appearance of the O-C-O bending vibrations in the 873 cm^−1^ band, which were due to carbonization in the slurry and due to the production of CO_3_^2−^ (Huo et al. 2021).

By combining Table 5 and Table 6, it can be seen that for the same curing time, the C-S–H transmittance values of B_4_, C_4_, and D_4_ were greater than that of A. The larger the transmittance was, the higher the peak strength, the better the hydration reaction, and the greater the amount of hydration products (C-S–H) (Li et al. 2020). In samples of the same age, the C-S–H transmittance of C_4_ and D_4_ was higher than that of A and B_4_. Table 6 shows that there was no detectable free Si–OH in B_4_ and D_4_, indicating that their hydration products had high degrees of polymerization and better hydration effects.

**Table 5:** The C-S–H transmittance (%) of samples at 3, 14, and 28 days

Group	3 days	14 days	28 days
A	33.14	29.61	11.19
B_4_	41.73	24.43	39.41
C_4_	46.87	40.94	49.88
D_4_	47.66	34.99	43.93

**Table 6:** The Si–OH transmittance (%) of samples at 3, 14, and 28 days

Group	3 days	14 days	28 days
A	43.16	41.78	42.26
B_4_	–––	–––	–––
C_4_	68.76	62.56	54.59
D_4_	–––	–––	–––

–––, no obvious band

### TG-DSC analysis

**Fig. 10:**
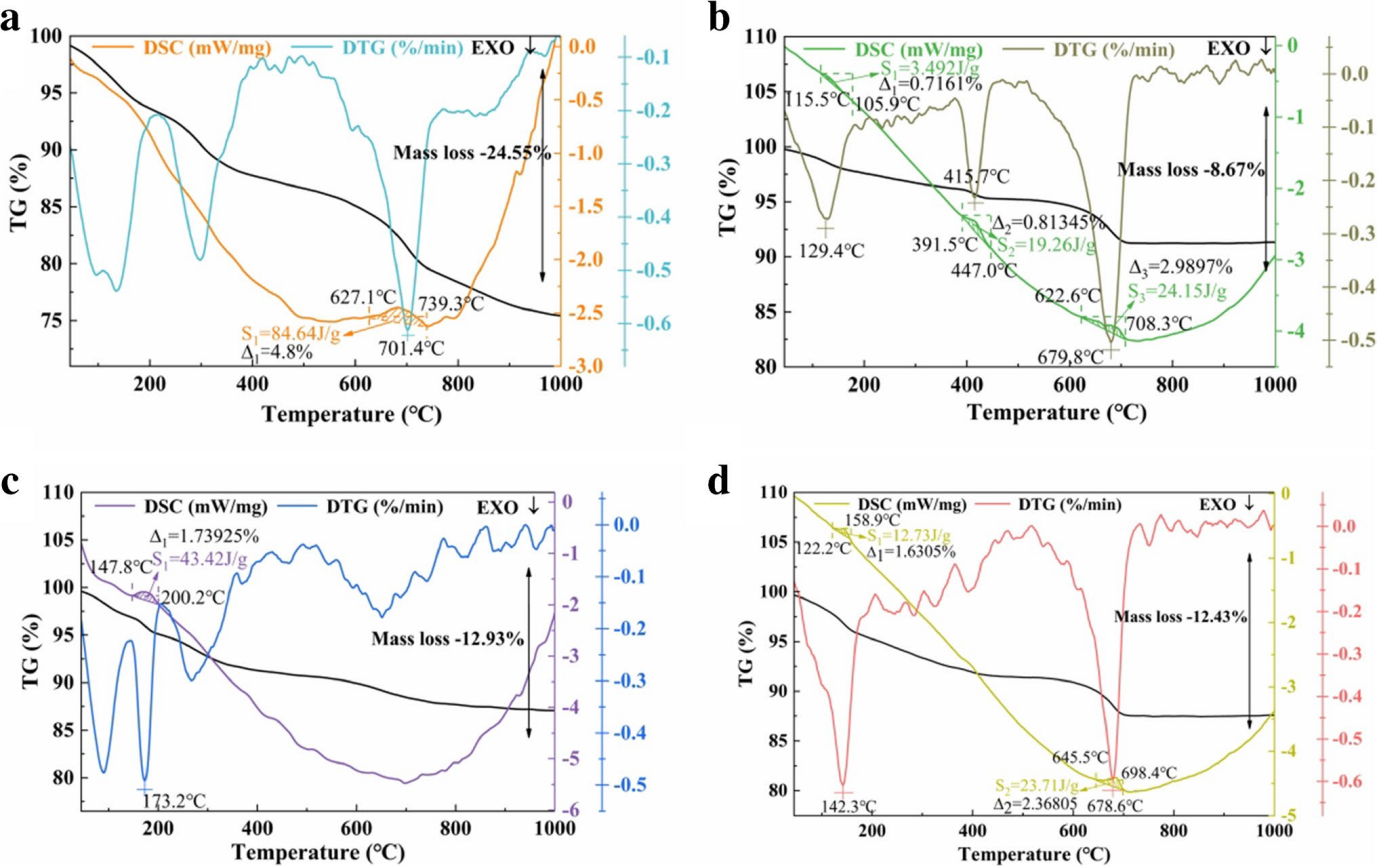
Comprehensive thermal analysis of samples at 14 days (**a** RM-based cementitious sample A, **b** 1000 °C RM thermal activation B_4_, **c** 1000 °C RM thermoalkali activation C_4_, **d** 1000 °C RM thermocalcium activation D_4_, **S** calorific value change, Δ mass loss)

The TG-DSC curves at 45 ~ 1000 °C of samples A, B_4_, C_4_, and D_4_ cured for 14 days in Fig. 10. Overall, the quality variation trend of the four group samples was similar, and the mass loss was 24.55%, 8.67%, 12.93%, and 12.43%, respectively. According to the analysis of Fig. 6 and Fig. 9, the weight loss before 400 °C was mainly caused by the evaporation of free water in hydration products and the removal of bound water; the weight loss at 400 ~ 600 °C was mainly caused by the thermal decomposition of Ca(OH)_2_; the weight loss at 600 ~ 800 °C was mainly affected by the thermal decomposition of CaCO_3_ produced by the cementitious samples during the cured period.

The mass loss of the cementitious sample was proportional to the hydration product content (Liu et al. 2018; Shao et al. 2020). By comparing the four groups samples, it was found that the obvious mass change of sample A only occurred in the range of 600 ~ 800 °C, and the mass loss in this temperature range was greater than that in the other samples, indicating that there was more CaCO_3_ in sample A. More CaCO_3_ production improved the carbonation properties, the worse the mechanical properties, which was consistent with the results of the study in Fig. 5. The mass loss of 400 ~ 600 °C only appeared in sample B_4_, which indicated that the content of Ca(OH)_2_ in the 1000 °C RM thermal activation cementitious sample was higher than that in other samples. Compare B_4_, C_4_, and D_4_, it could be seen that the mass loss at 45 ~ 400 °C was 1.52955%, 1.73925%, and 1.6305%, respectively, demonstrating that there were more growing hydration products in the RM thermoalkali activation cementitious materials. With the extension of curing time, the hydration products would be more and more, showing the late strength of the cementitious sample. The conclusion was consistent with Fig. 5, indicating that the activation of RM by thermoalkali activation at 1000 °C could promote the hydration reaction process. After the RM was thermally activated, most of the minerals would be released. The RM after the activation of thermoalkali and thermocalcium could generate more aluminum silicate, which was beneficial to improve the hydration process of cementitious materials.

### Hydration product formation process

**Fig. 11:**
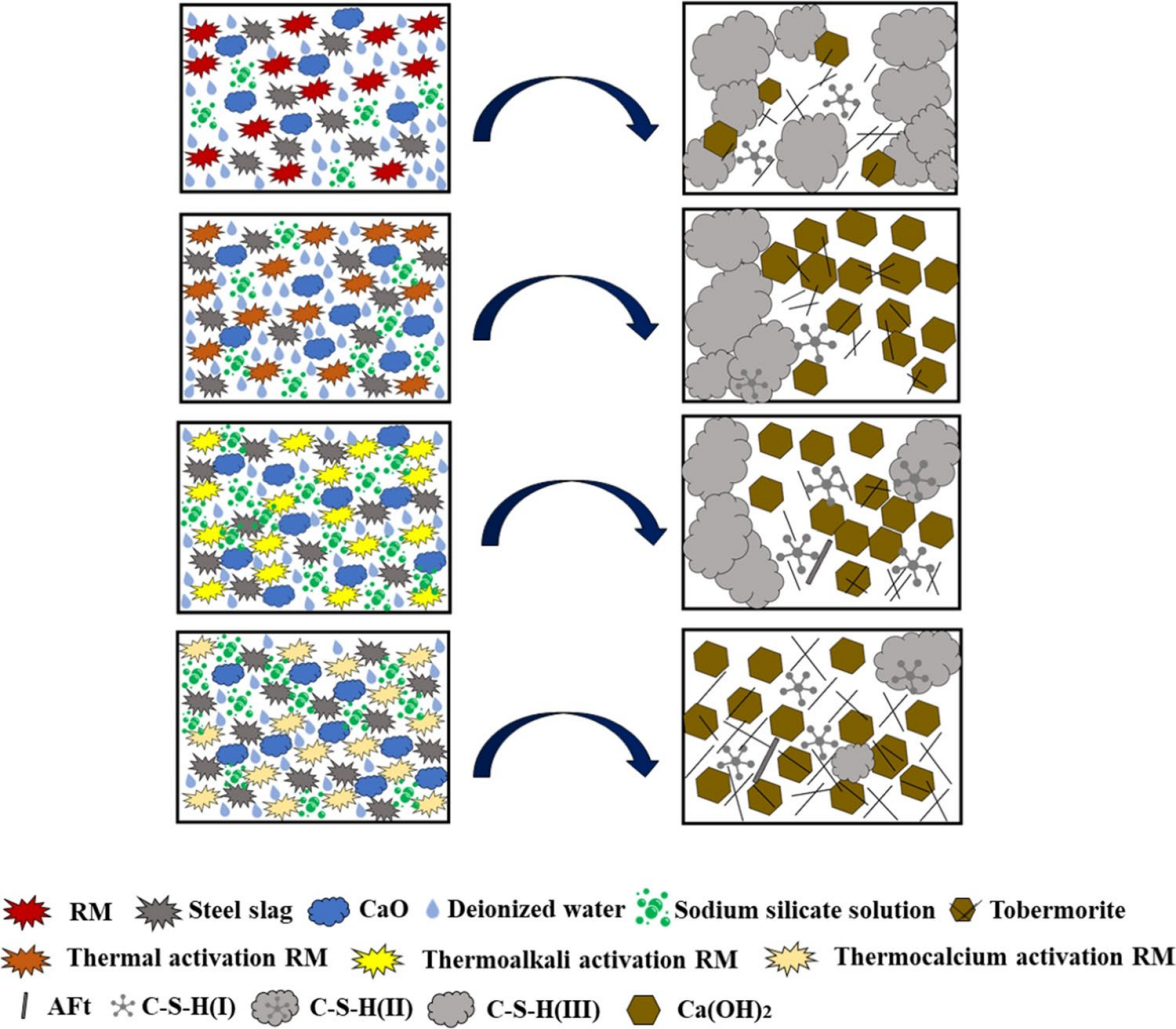
Hydration product formation process of RM with different pretreatments

Figure 11 shows the formation process of the hydration products with different pretreatment RM (Cheng et al. 2019). The hydration products of the samples made by different pretreatment methods were mainly Ca(OH)_2_, tobermorite, C-S–H, and AFt.

With a change in the pretreatment method, the morphology and distribution state of hydration products changed. The hydration products produced by the samples prepared from RM raw materials were mainly in the form of opaque clusters of C-S–H(III) and lamellar layers of interwoven tobermorite. Nevertheless, the hydration products in the samples prepared from thermally activated RM were mainly in the form of lamellar Ca(OH)_2_ wrapped with short curvilinear clouds of clustered C-S–H(II). Further investigation revealed that the hydration products in the thermoalkali-activated RM preparation samples again formed only a few short rod-like AFt; the reason was that under the conditions of surface energy release, there was not enough sulfate to react with aluminate and water contact to form AFt (Han et al. 2015; Tydlitát et al. 2014), while the hydration products in the thermocalcium-activated RM preparation samples had more tobermorite formed by interwoven needle-like lamellae. The above studies illustrated that the proper thermal treatment of RM raw materials could enhance Ca(OH)_2_ production, which indicated that a single temperature could activate the Ca-based components of RM to participate in the hydration reaction. The thermoalkali treatment of RM raw materials could induce the conversion of opaque agglomerates of coarse C-S–H(III) to C-S–H(II) due to thermoalkali activation could improve the activity of the original Ca–Si matrix of RM, providing stable alkaline conditions that could promote the process of the hydration reaction of the material. Thermocalcium treatment of the RM raw materials showed that the hydration products were mainly C-S–H, tobermorite, and AFt, which indicated that more tobermorite could be produced in RM treated by the addition of appropriate amounts of calcium matrix components with heating, and the samples prepared by thermal calcium treatment of RM had higher mechanical properties, which was consistent with the results of the analysis in Fig. 5.

### ICP analysis of elements

**Fig. 12:**
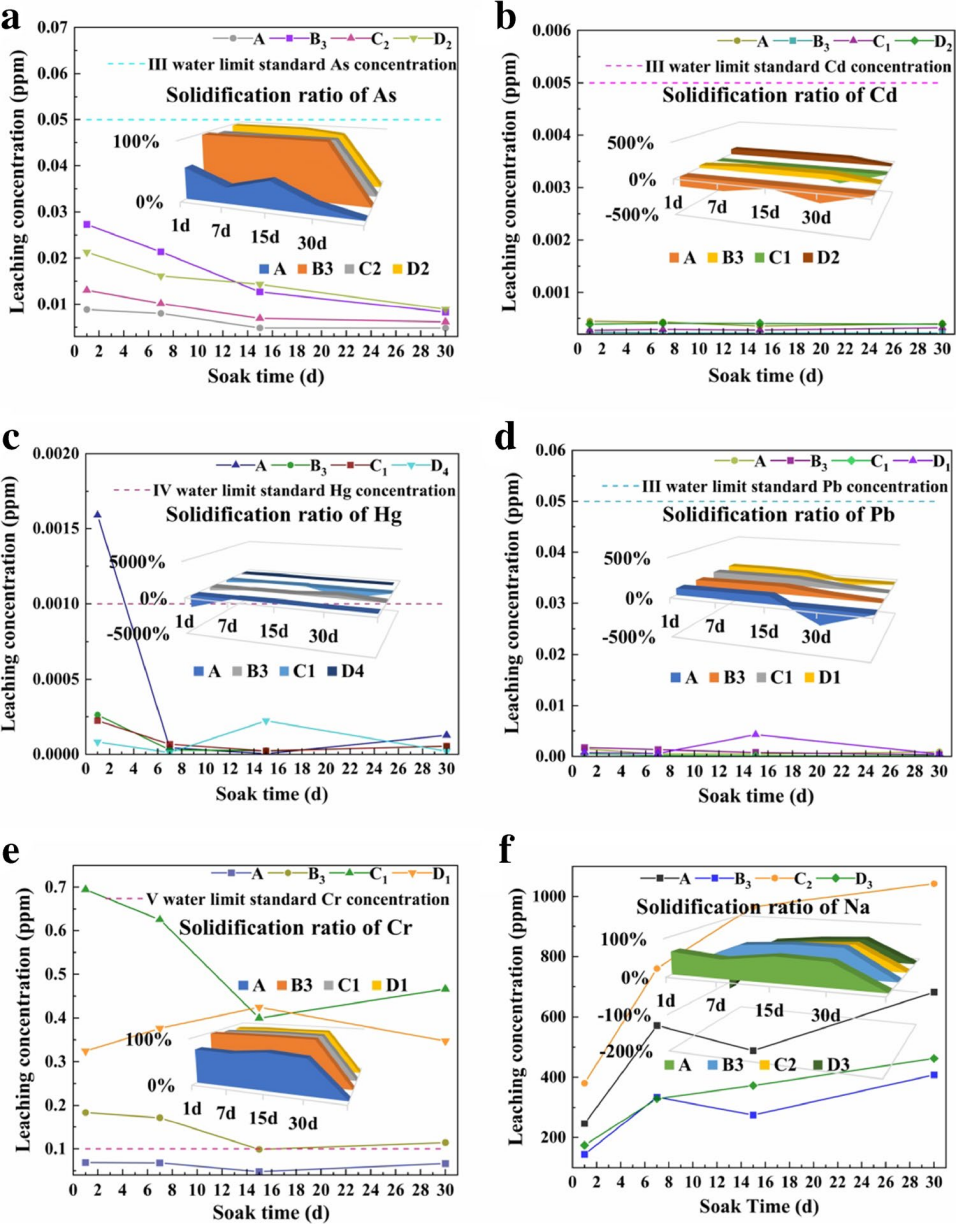
Solidification ratio and leaching concentration of elements for soaking 1, 7, 15, and 30 days of 28-day samples ( **a** As, **b** Cd, **c** Hg, **d** Pb, **e** Cr, **f** Na)

The pretreated RM and samples which cured at 28 days were soaked for 1 day, 7 days, 15 days, and 30 days according to the acetic acid buffer solution method (HJ/T 300–2007), and the solidification ratio was calculated according to the elemental concentration detection in the immersion solution, as shown in Fig. 12. RM had a large specific surface area, which could adsorb heavy metals to a certain extent. The silica-oxygen tetrahedra and aluminum-oxygen tetrahedra in the RM-based cementitious material formed aluminum silica gelation to make the sample denser, which acted as a wrapper for Cu^2+^ and reduced its migration rate (Wen et al. 2022). Many studies had claimed that HCl could undergo replacement reactions with CaO, causing the dissolution of Na, and K in RM and producing a large number of hydrotalcite-like compounds, forming hydroxy carbonates (Luo et al. 2017). Titanium dioxide waste acid could also dissolve RM to obtain calcium chalcocite and hard gypsum with the disrupted crystal lattice. Where the leaching process of K from RM was controlled by external diffusion (Zhu et al. 2022). In the presence of these strong acids, the release rate of heavy metals from RM increased. In contrast, heavy metals in RM were not easily moved under natural environmental conditions, even under moderately acidic or reducing conditions (Rubinos and Barral 2013). Therefore, in this study, the acetate buffer solution was chosen to simulate the presence of natural environmental acid rain in the atmosphere. The solidification ratio of each element in the samples was calculated according to the following formula:

5 $$m=x/y$$

6 $$k=1-m$$

where *x* is the leaching concentration of each element in the sample (ppm); *y* is the element leaching concentration of different thermally activated RMs corresponding to different samples (ppm); *m* is the dissolution ratio (%); and *k* is the solidification ratio (%).

When *k* > 0, the amount of solidified element was greater than the amount leached, which further indicated that the effect of element solidification was better; however, when *k* < 0, the opposite effect was observed.

Since the samples corresponding to the stable states of the leached and solidified elements were different, Fig. 12 shows the samples that best corresponded to the leached and solidified states of different elements. Figure 12 a–d shows the leaching and solidification conditions for As, Cd, Hg, and Pb in the samples. The leached concentration of elements in the sample was below the limit value for class III and class IV surface waters. B_3_ displayed a good solidification effect of these elements, and the solidification ratio of As was 98.76%. Figure 12 e shows the leaching and solidification of Cr in the samples. The concentrations of Cr leached from A and B_3_ were lower than the limit of class V for surface waters, in which B_3_ had the best solidification effect and the solidification ratio of Cr was 99.52%. Due to the alkaline nature of RM, after thermal activation, thermal alkali activation, and thermal calcium activation, the alkalinity further increased and the pH of RM was increased; Cd, Pb, and Cr formed low solubility hydroxides which were difficult to be leached. Meanwhile, Ca and Al in the hydration products could be replaced by Cd, Pb, and Cr stabilized in the hydration products (Liu et al. 2009), which made the leaching concentration of heavy metals decrease. Moreover, the thermal activation temperature of RM had a great influence on the leaching concentration of heavy metals. The above research showed that the samples prepared by the thermal treatment of RM and SS at 900 °C had a better solidification effect on heavy metals, while the samples activated by thermoalkali and thermocalcium had the best solidification effect on heavy metals at 600 °C. The adsorption rate of Hg on RM was fast and its kinetic process was described by Ho’s pseudo-second-order equation (Rubinos and Barral 2015), and there was a relationship between chemisorption and temperature. By analyzing, when the temperature for thermocalcium-activated RM reached 1000 °C, Hg had a low leaching concentration.

Figure 12 f shows the leaching and solidification status of Na in the samples. The leached concentration of Na showed an increasing trend because the thermal treatment of RM increased the activity of Ca, Al, and Si, and the thermoalkali treatment and thermocalcium treatment further increased the alkalinity of the samples. The samples with lower leaching concentrations were B_3_ and D_3_, which indicated that the samples treated with thermal treatment and thermocalcium at 900 °C had more stable alkalinity, implying that 900 °C was a better pretreatment temperature.

### Specific binding energy

**Fig. 13:**
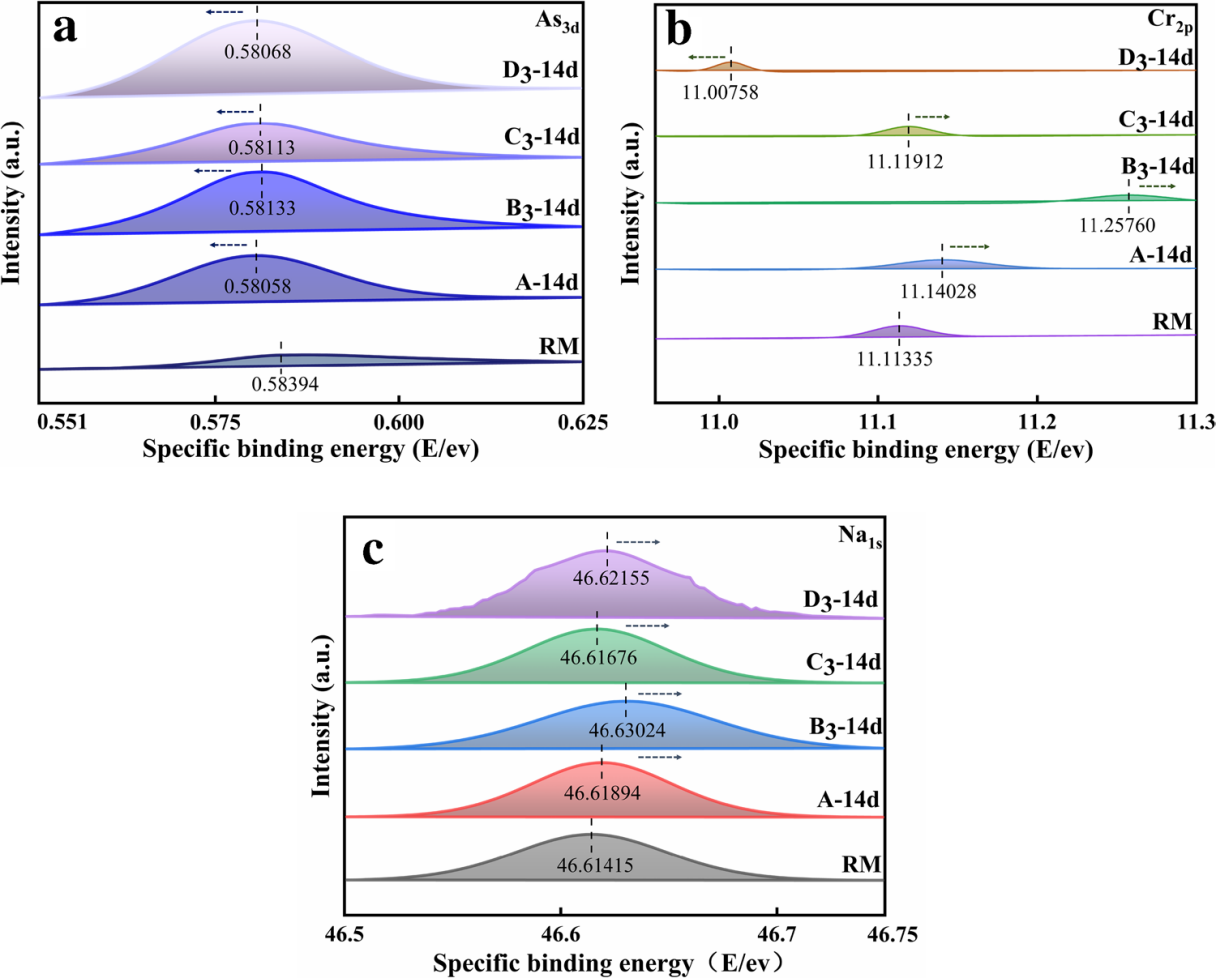
Specific binding energy of Na_1s_, As_3d_, and Cr_2p_ in RM and samples at 14 days

The larger the specific binding energy is, also known as the average binding energy (SBE), the stronger the binding of atoms and the more stable the structure. Figure 13 shows the change in the specific binding energy of As_3d_, Cr_2p_, and Na_1s_ for RM, A, and the samples of RM with different pretreatments at 900 °C.

By comparing the RM and A, it can be seen that the SBE of Cr_2p_ and Na_1s_ in the sample was greater than that in the RM, which indicated that Cr_2p_ and Na_1s_ were present in a more solid form in the sample, which was due to the laminar structure of the hydration product C-S–H that could adsorb or encapsulate the Cr (Zhang et al. 2005), while As_3d_ was in a readily leached state. Relative to A, further thermal activation of RM at 900 °C, thermal alkali activation, and thermal calcium activation, showed that the SBE of As_3d_, Cr_2p_, and Na_1s_ was significantly higher in B_3_.

However, the atomic SBE in the thermal alkali-activated and thermal calcium-activated samples did not change much or even decreased in comparison with A, which indicated that thermal activation at 900 °C had a positive effect on the solidification of heavy metals and alkali substances and reflected that the applied alkali and calcium had an interfering effect on the heavy metals and alkali elements in the samples, and the amount and temperature of the applied alkali and calcium need to be further studied.

## Conclusions

In this work, RM, three kinds of preactivated RM were combined with SS to prepare cementitious materials, respectively. The mechanical properties, hydration reactions, and environmental safety of the cementitious materials were studied, and the investigation yielded the following results.

- Mechanical properties

The thermal-activated RM showed much higher mechanical properties than the original RM. The 14-day cementitious samples could achieve a flexural strength greater than 3.0 MPa for a single sample when the RM was thermally or thermocalcium activated. Meanwhile, the 28-day thermoalkali-activated RM cementitious samples showed similar flexural strength. The optimal preactivated temperatures of different preactivated RM were different, and the thermoalkali-activated RM had an optimal preactivated temperature at 1000 °C, while the optimal preactivated temperature for both thermal activated and thermocalcium activated RM was 900 °C, because the proper increase in temperature was beneficial to the activation of the gelling factor at higher alkali content. The mechanical properties of 900 °C thermocalcium-activated RM cementitious materials were better, because the proportion of Ca matrix in the cementitious hydration products C-S–H played an important role in the nucleation and growth of hydration products. Additionally, the increase in temperature could stimulate the activity of potential cementitious factors in RM, resulting in there were more hydration products with uniform distribution, thus showing excellent mechanical properties.

(b) Hydration mechanisms

The cementitious materials prepared by original RM, preactivated RM, and SS, respectively, had similar hydration products which consist of tobermorite, C-S–H, Ca(OH)_2_, and AFt, with differences in the microstructures of the hydration products depending on the different preactivated methods. The hydration products of un-activated RM-based cementitious materials were ungrown fine lamellae and needles of tobermorite; the hydration products of thermally activated and thermoalkali-activated RM-based cementitious materials were dominated by lamellar Ca(OH)_2_, and the grown tobermorite in the form of interwoven lamellae and needles was mainly hydration products of thermocalcium-activated RM-based cementitious materials. The variation of the above morphology was related to the ratio of Ca/Si in the cementitious material, and the ratio of Ca/Si should be adjusted within 5 ~ 12 considering the hydration performance of the cementitious samples.

(iii) Environmental risks

Compared with the original RM-based cementitious material, the leaching concentration of heavy metal elements in the preactivated RM-based cementitious material fluctuates in a small range, because temperature and additional substances affect the combination state of heavy metal elements and hydration products. Cd, Pb, and Cr replaced Ca, and Al in hydration products to improve the solidification ratio while Hg was adsorbed on hydration products to achieve the solidified effect, and these fluctuations still satisfied the surface water limit. The leaching concentrations of As, Cd, Hg, Pb, and Cr in 900 °C thermally activated RM-based cementitious samples were lower than the limits of class III or class IV surface waters; 600 ~ 800 °C thermoalkali activation had a good effect on the solidification of As, Cd, Hg, Pb, and Cr. 600~800℃ thermocalcium activation also had an ideal activation effect on the solidification of As, Cd, Pb, and Cr; however, a better effect of solidification of Hg required 1000 °C thermocalcium activation pretreatment, which was related to the adsorption of Hg on RM was affected by temperature. The leaching concentration of alkali substances in the thermal and thermocalcium RM-based cementitious material was lower than that in the original RM-based cementitious material, which indicated that the temperature not only increased the pH of the sample, but also improved the binding effect of alkali substances and hydration products.

## Data Availability

Data and materials are contained within the article.
